# A Comparative Study of the Chemical Composition and Skincare Activities of Red and Yellow Ginseng Berries

**DOI:** 10.3390/molecules29204962

**Published:** 2024-10-20

**Authors:** Yu-Dan Wang, Lu-Sheng Han, Gen-Yue Li, Kai-Li Yang, Yan-Long Shen, Hao Zhang, Jian-Feng Hou, En-Peng Wang

**Affiliations:** 1 Jilin Ginseng Academy, Changchun University of Chinese Medicine, Changchun 130117, China; 22203070305@stu.ccucm.edu.cn (Y.-D.W.); 22203560007@stu.ccucm.edu.cn (L.-S.H.); 22203560012@stu.ccucm.edu.cn (G.-Y.L.); 22203560024@stu.ccucm.edu.cn (K.-L.Y.); 2 College of Innovation and Entrepreneurship, Changchun University of Chinese Medicine, Changchun 130117, China; shyl91@163.com; 3 Institute of Special Animal and Plant Sciences CAAS, Changchun 130112, China; zhanghaoscience@163.com; 4 Shiqi Biological R&D Centre (Suzhou Industrial Park) Co., Ltd., Suzhou 215125, China; jeffery_hou@sikibio.com

**Keywords:** ginseng, ginseng berry, chemical composition, anti-aging, anti-UVB, anti-melanin, whitening

## Abstract

This study was conducted to investigate the differences in chemical composition between red (RGBs) and yellow ginseng berries (YGBs) and their whitening and anti-aging skincare effects. The differences in the chemical composition between RGB and YGB were analyzed by ultra-high-performance liquid chromatography tandem quadrupole electrostatic field orbit trap mass spectrometry (UHPLC-Q-Exactive-MS/MS) combined with multivariate statistics. An aging model was established using UVB radiation induction, and the whitening and anti-aging effects of the two ginseng berries were verified in vitro and in vivo using cell biology (HaCaT and B16-F10 cells) and zebrafish model organisms. A total of 31 differential compounds, including saponins, flavonoids, phenolic acids, and other chemical constituents, were identified between the two groups. Superoxide dismutase (SOD) activity was more significantly increased (*p* < 0.05) and malondialdehyde (MDA) content was more significantly decreased (*p* < 0.01) in RGB more than YGB induced by UVB ultraviolet radiation. In terms of whitening effects, YGB was more effective in inhibiting the melanin content of B16-F10 cells (*p* < 0.01). The results of zebrafish experiments were consistent with those of in vitro experiments and cell biology experiments. The DCFH fluorescence staining results revealed that both ginseng berries were able to significantly reduce the level of reactive oxygen species (ROS) in zebrafish (*p* < 0.01). Comparison of chemical composition and skin care activities based on RGB and YGB can provide a theoretical basis for the deep development and utilization of ginseng berry resources.

## 1. Introduction

*Panax ginseng* C. A. Mey., one of the traditional Chinese herbs, has a variety of pharmacological actions and therapeutic effects [1]. Ginseng contains saponins, polysaccharides and active peptides, which have a variety of active effects including antioxidant, anti-aging and whitening [2,3]. The aboveground parts of ginseng include roots, stems, leaves, flowers and berries. Ginseng berry is the main production area in Jilin Province, China, and its production is abundant, which can reach more than 800 tons per year. Modern pharmacology has shown that ginseng berries can improve cognitive function, memory, hypoglycemia, anti-inflammatory, and many other pharmacological activities [4,5,6,7,8,9]. In recent years, a variety of chemical components have been isolated from ginseng berries, including ginsenosides, polysaccharides, amino acids, and inorganic elements [6,10]. The relevant literature suggests that ginseng-berry-mediated metal nanoparticles can be utilized as novel cosmetic materials for skin care [11]. Heat-treated ginseng berries were able to enhance the conversion of rare ginsenosides Rk1, Rg5, and Rg3 and inhibit melanogenesis by inducing autophagy [12]. The (+)-syringaresino in ginseng berries accelerates cell proliferation and has the potential to promote skin healing [9]. Therefore, ginseng berries have the potential to be developed as a cosmetic ingredient.

The combination of aging and UVB radiation aggravates cell metabolism, which in turn produces melanin deposits [13,14]. The pursuit of natural cosmetic ingredients has become mainstream in today’s society. Natural berries and seeds are rich in flavonoids, vitamins, carotenoids, anthocyanins, and many other antioxidants [15]. Red goji berries and black goji berries (Lycium fruits) contain high levels of polyphenols with strong antioxidant properties [16]. It has the effect of relieving oxidative stress, anti-radiation, and anti-aging [17]. The colors of mature ginseng berries are classified as red, yellow, pink, orange, etc. YGB is a new ginseng variety endemic to Jilin Province, China, and is a pure recessive mutant of the traditionally cultivated RGB [10]. Currently, there are few basic studies on the efficacy components of the germplasm resources of the new YGB cultivar. The differences in chemical composition and related activities of ginseng fruits of different colors are inconclusive.

Ultra-high performance liquid chromatography tandem quadrupole electrostatic field orbit trap mass spectrometry (UHPLC-Q-Exactive-MS) coupled with multivariate statistical analysis has been widely used in the field of pharmaceuticals and food development with a high resolution, sensitivity, rapidity, and throughput [18]. In this paper, we intend to analyze the differences in the chemical composition of two ginseng berry colors and initially elaborate the differences in anti-aging and skin-whitening activities of different berry colors to provide a scientific basis for the development and utilization of ginseng berry resources.

## 2. Results

### 2.1. Differential Compound Analysis of Ginseng Red and Yellow Berries by UHPLC-Q-Exactive-MS

**Figure 1:**
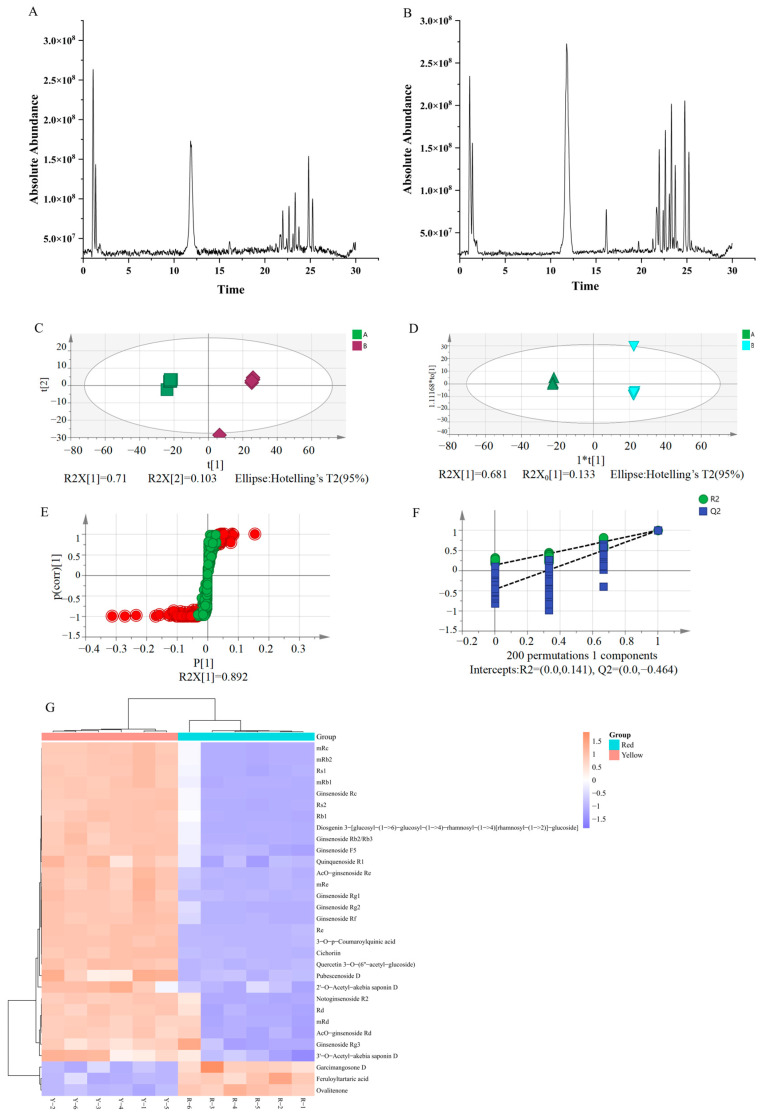
Chemical composition of RGB and YGB based on UHPLC-Q-Exactive-MS combined with multivariate statistical analysis. (**A**) TIC of RGB. (**B**) TIC of YGB. (**C**) PCA score plots. (**D**) OPLS-DA score plots. (**E**) S-plot score plots. (**F**) Permutation test chart. (**G**) Heat map analysis of RGB and YGB. Note: The total ion chromatograms (TIC); principal components analysis (PCA); orthogonal partial least squares discriminant analysis (OPLS-DA)

As shown in Figure 1A,B, the total ion chromatogram(TIC) of the ginseng berry sample was well separated and had a high response value in the negative ion mode. The RGB and YGB have different contents and types of chemical components. The multivariate statistical analysis revealed that the PCA showed that the total variance explained of the samples was 81.6%, indicating that the model was stable. The analysis of OPLS-DA results showed that the total-variance-explained rate of the samples was 81.2%, which indicated that the reliability of the model was good and the samples could be clearly distinguished between the two groups. The OPLS-DA was further tested using a permutation test. The number of randomized tests was set to 200, and the results showed that the intercepts of R^2^ and Q^2^ with the Y-axis were 0.473 and −0.257, respectively, indicating that the model had good predictive ability. As shown in Figure 1F, the metabolites with significant differences (*VIP* > 1) were screened using S-plot diagrams. This indicated that the model was well separated and stable, and that the two fruit-colored ginseng berries had large differences in their chemical compositions. The results of cluster analysis showed that YGB and RGB could be clearly classified into 2 groups. As shown in Figure 1G, the high expression zone of differential compounds in YGB totaled 28 chemical components, mainly ginsenosides and phenolic acids. Based on the importance values of the projected variables (*VIP*) in the OPLS-DA model, combined with the t-test and the multiplicity of differences (*FC*), the differential compounds in the two berry colors were screened (*VIP* > 1, *p* < 0.05, *FC* > 2). Based on the *m*/*z* values of the single isotope peaks of the differential compounds, the ion fragmentation and retention time were imported into HMDB and PubChem databases for matching, and a total of 31 differential compounds were identified, as shown in Table 1. Based on the obvious hybridization between RGB and YGB germplasm resources, there are differences in the content and nature of their chemical components, and their pharmacological activities have significant differences.

### 2.2. Determination of Antioxidant Capacity of Red and Yellow Ginseng Berry

**Figure 2:**
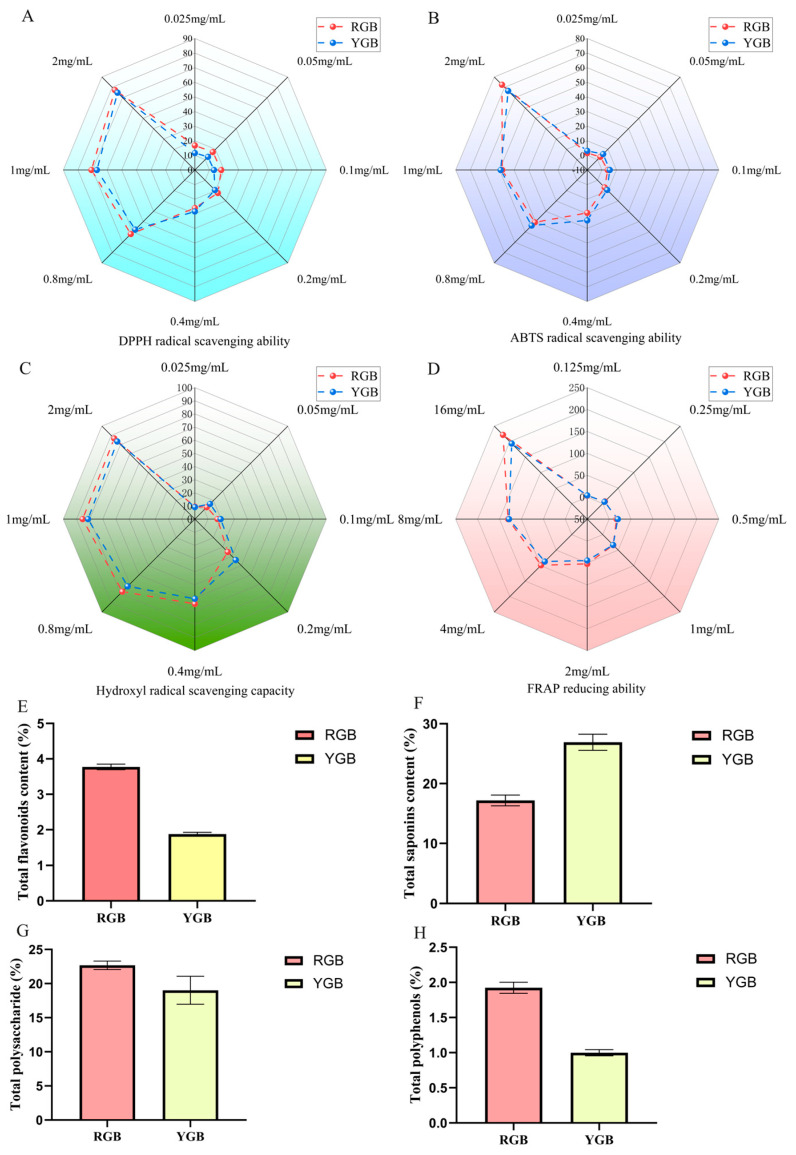
Comparison of in vitro antioxidant capacity and content determination of ginseng berries. (**A**) Measurement of DPPH-scavenging capacity of RGB and YGB. (**B**) Measurement of ABTS scavenging capacity of RGB and YGB. (**C**) Measurement of hydroxyl-radical-scavenging capacity of RGB and YGB. (**D**) Measurement of FRAP-reducing ability of RGB and YGB. (**E**) Total flavonoids content of RGB and YGB. (**F**) Total saponins content of RGB and YGB. (**G**) Total polysaccharide RGB and YGB. (**H**) Total polyphenols content of RGB and YGB.

For calculating the FRAP reduction ability, the following regression equation was used: Y = 3.762 x + 0.1627 (*r* = 0.9994). The antioxidant capacity of the samples was assayed using Fe^2+^ to generate a blue–violet complex with TPTZ under acidic conditions. As shown in Figure 2A–D, ginseng berries were found to have greater free-radical-scavenging ability with increasing drug concentration, as measured by a total of four antioxidant indicators DPPH/ABTS/OH/FRAP. The IC_50_ is commonly used to evaluate the antioxidant activity, and the smaller the IC_50_ value, the stronger its antioxidant property [19]. Both RGB and YGB possessed strong antioxidant activity, and the antioxidant capacity of RGB was found to be slightly stronger than that of YGB by IC_50_.

### 2.3. Comparative Quantification of Chemical Composition of Ginseng Berry

For calculating the total flavonoids content, the following regression equation was used: Y = 7.12x+ 0.0500 (*r* = 0.9999). The Y-value is the absorbance value of SGW, while the X-value is calculated using the same formula used to obtain the total flavonoids content.

For calculating the total saponins content, the following regression equation was used: Y = 11.23x+ 0.0247 (*r* = 0.9998). The Y-value gives the absorbance value of SGW, and the X-value is calculated using the same formula used to obtain the total saponins content.

For calculating the total polysaccharide content, the following regression equation was used: Y = 1.6769x+ 0.1166 (*r* = 0.9999). The absorbance values were substituted into the standard curve as Y-values, and the X-values were calculated to obtain the total polysaccharide content.

For calculating the total polysaccharide content, the following regression equation was used: Y = 0.028x+ 0.1677 (*r* = 0.9997). The absorbance values were substituted into the standard curve as Y-values, and the X-values were calculated to obtain the total polyphenols content.

As shown in Figure 2E–G, the ginseng berry constituents were found to be significantly different by a total of four content determinations of total flavonoids/total saponins/total polysaccharides/total polyphenols. As shown in Table 2, total ginsenosides of YGB ginseng were found to be higher than RGB by chemical quantification, which is consistent with previous results. The statistical results of non-targeted metabolomics in Table 1 revealed that YGB contained a variety of rare human saponins, such as Ginsenoside Rf, Ginsenoside F3, and Ginsenoside Rc. This was correlated with the ginsenoside species and content. The variation in fruit color was mainly affected by the cumulative content of flavonoids, anthocyanosides, and carotenoids, including red, pink, and reddish-purple [20]. In addition, the total flavonoids, total polysaccharides, and total polyphenols contents were high in RGB. The reason for this may be due to the influence of fruit color.

### 2.4. Evaluation of Red and Yellow Ginseng Berries’ Tyrosinase Inhibition Rate

**Figure 3:**
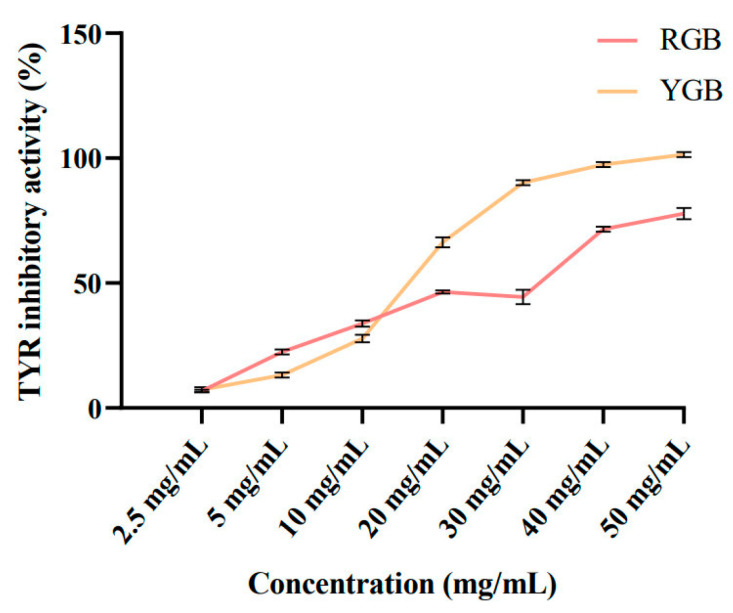
Comparison of ginseng berries on tyrosinase activity.

Tyrosinase is a key enzyme in melanogenesis and a major factor in the enzymatic browning of fruits and vegetables [21]. Tyrosinase converts L-tyrosine to L-3,4-dihydroxyphenylalanine (L-DOPA), which is converted to dopaquinone, and then undergoes a series of reduction and oxidation reactions to become melanin [22]. The smaller IC_50_ value represents the stronger tyrosinase inhibition ability of the sample. As shown in Figure 3, the IC_50_ value of RGB was 21.09 mg/mL, and the IC_50_ value of YGB was 14.67 mg/mL. The oxidative pathway of tyrosine to produce melanin can be blocked by ginsenosides Rb1 and Rg1, thereby reducing melanin production and contributing to skin-whitening effect. YGBs are more capable of inhibiting melanogenesis than RGBs, which may be related to the high content of ginsenosides in YGBs. The results of chemical composition identification revealed that YGBs contain rare ginsenoside Rg3, ginsenoside Rg2, and ginsenoside Rb2. Through RGB transformation, green ginseng berries are enriched with ginsenoside Rg2, which is more effective in inhibiting melanin production through the NF-κB pathway [23]. Ginsenoside Rb2 was effectively isolated from ginseng berries, which can be used as a potential whitening antioxidant to inhibit melanin synthesis in vivo and in vitro to achieve whitening efficacy [24].

### 2.5. Determination of HaCaT and B16-F10 Cell Activity by Red and Yellow Ginseng Berries

#### 2.5.1. Screening of Red and Yellow Ginseng Berry Concentration and UVB Radiation Dose

**Figure 4:**
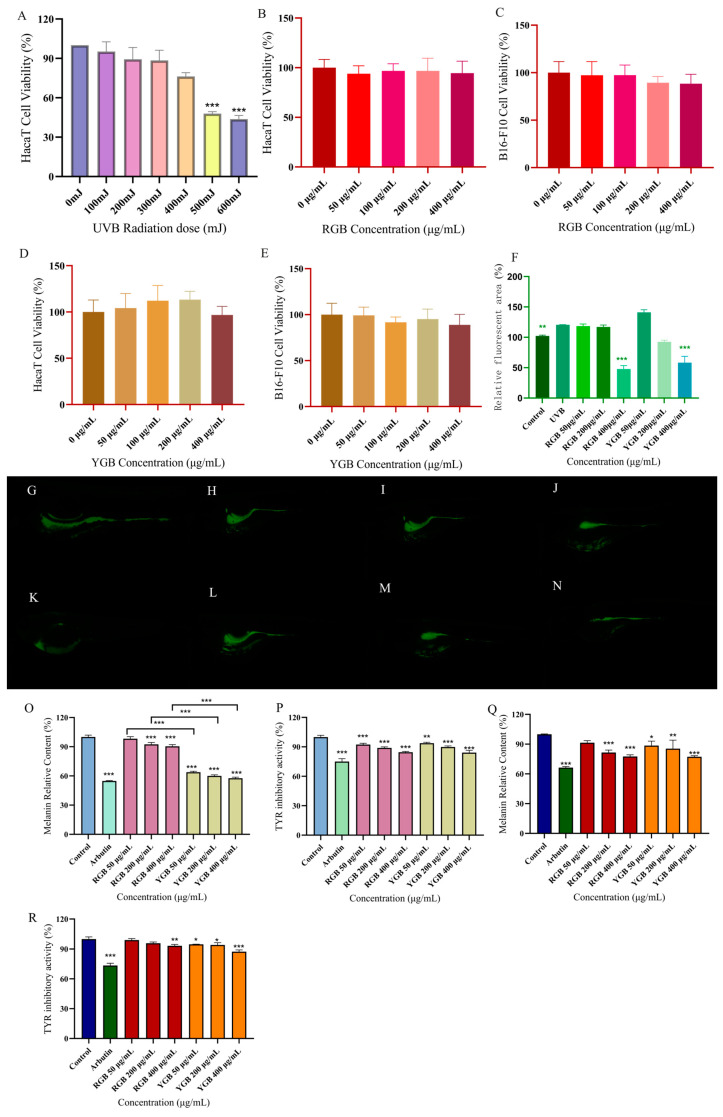
Comparing the anti-aging and whitening effects of ginseng berries of different fruit colors. (**A**) UVB radiation dose to HaCaT cells. (**B**) Red berries HaCaT cytotoxicity assay. (**C**) Red berries B16-F10 cytotoxicity assay. (**D**) Yellow berries HaCaT cytotoxicity assay. (**E**) Yellow berries B16-F10 cytotoxicity assay. (**F**) Statistical analysis of RGB and YGB fluorescence areas. (**G**) Control group. (**H**) UVB group. (**I**) 50 μg/mL RGB. (**J**) 200 μg/mL RGB. (**K**) 400 μg/mL RGB. (**L**) 50 μg/mL YGB. (**M**) 200 μg/mL YGB. (**N**) 400 μg/mL YGB. (**O**) Relative melanin content in B16-F10 cells. (**P**) Relative tyrosinase content in B16-F10 cells. (**Q**) Relative melanin content in zebrafish (**R**) Relative tyrosinase content in zebrafish. Note: * p < 0.05; ** p < 0.01; *** p < 0.001. DCFH-DA Reactive Oxygen ROS Fluorescent Probe (2′,7′-Dichlorodihydrofluorescein Diacetate).

As shown in Figure 4, cell survival showed a decreasing trend with increasing UVB radiation dose. The survival rate was lower than 50% at 500 mJ (*p* < 0.001), and 400 mJ was chosen for subsequent experiments. In Figure 4B–D, the two-fruit-colored samples acted on HaCaT and B16-F10 cells, respectively, and there was no significant difference in the range of 0–400 μg/mL (*p* > 0.05), with low cytotoxicity. Therefore, 50 μg/mL, 200 μg/mL, and 400 μg/mL of ginseng berries were selected for subsequent experiments.

#### 2.5.2. Determination of Antioxidant Damage Capacity of Ginseng in Different Concentrations of Red and Yellow Ginseng Berry

SOD activity, MDA content, CAT activity, and GSH-P_X_ activity were determined according to the kit. The results are shown in Table 3. Compared with the blank group, SOD activity, CAT activity, and GSH-P_X_ activity were significantly decreased in the UVB model group (*p* < 0.001); MDA content was significantly increased (*p* < 0.001). This revealed that UVB radiation induced oxidative stress in HaCaT cells, further indicating that the model was successfully modeled with significant differences. Compared with the model group, RGB and YGB were able to increase SOD activity, CAT activity, GSH-P_X_ activity (*p* < 0.001) and decrease MDA content (*p* < 0.001), showing a certain quantitative effect relationship, indicating that ginseng berries were able to reduce UVB-radiation-induced oxidative stress and possessed a significant capacity to resist oxidative damage. However, 400 μg/mL RGB had more potential antioxidant and anti-aging effects than YGB, and there was a significant difference between them.

#### 2.5.3. Evaluation of Whitening Activity of B16-F10 Cells by Red and Yellow Ginseng Berries

In Figure 4O, there was a tendency for the relative content of melanin to decrease in all groups compared with the blank group of B16-F10 cells. The relative melanin content in the arbutin group amounted to 54.8 ± 0.5% (*p* < 0.001) compared with the blank group. The melanin content of B16-F10 cells in the 400 μg/mL RGB administration group was significantly reduced, and the relative melanin content amounted to 90.5 ± 1.7% (*p* < 0.001). The relative melanin content of B16-F10 cells in the 400 μg/mL YGB administration group reached 57.6 ± 1.2% (*p* < 0.001). This indicated that both RGBs and YGBs could significantly inhibit melanogenesis in B16-F10 cells in the concentration range of 50–400 μg/mL, and the inhibitory effects all showed a certain dose-dependence. At the same concentration, YGB had a significant difference (*p* < 0.001) compared with RGB, which was able to reduce melanin synthesis and had more whitening efficacy. Compared with the blank group, arbutin group showed a highly significant tyrosinase activity of 75.056 ± 2.846 (*p* < 0.001), the 400 μg/mL RGBs showed a tyrosinase activity of 84.545 ± 0.783, and the 400 μg/mL YGB showed a tyrosinase activity of 84.184 ± 2.240 (*p* < 0.001), and it decreased with the increase in concentration.

### 2.6. Evaluation of Anti-UVB Radiation and Whitening Efficacy of Red and Yellow Ginseng Berries in Zebrafish

#### 2.6.1. Determination of Inhibition of UVB-Induced ROS Oxidative Damage in Zebrafish by Red and Yellow Ginseng Berries

It was observed that zebrafish embryos developed well at concentrations of 0–400 μg/mL. In this concentration range, zebrafish embryo matter survival was 100% and not teratogenic. The most commonly used method to detect intracellular ROS levels using fluorescent staining is the DCFH-DA fluorescent probe method [25,26]. Intracellular ROS are able to oxidize non-fluorescent DCFH, and the intensity of green fluorescence is proportional to the level of ROS [27]. Zebrafish fluorescence area was calculated by Image J. After induction with 400 mJ of UVB radiation, the fluorescence intensity of zebrafish in the model group was significantly higher than that of the blank group, indicating the success of photoaging modeling (*p* < 0.01); after intervention with the drug, the ROS began to diminish in the zebrafish. Compared with the model group, the rate of ROS generation was significantly lower in the drug-administered group, indicating that the ginseng fruit mitigated oxidative stress damage by inhibiting ROS formation in zebrafish larvae.

#### 2.6.2. Evaluation of B16-F10-Cell-Whitening Efficacy by Different Concentrations of Red and Yellow Ginseng Berries

The developmental viability of zebrafish embryos at ginseng berry concentrations of 2.5–400 μg/mL had no toxic effects. As shown in Figure 4Q,R, the melanin content of zebrafish embryos was higher in the blank group, and all groups had a tendency to reduce the relative melanin content in vivo compared with zebrafish in the blank group. Compared with the blank group, the relative melanin content in the positive arbutin group amounted to 66.4 ± 1.1% (*p* < 0.001). Zebrafish embryos treated with RGB at a concentration of 400 μg/mL showed a significant decrease in melanin content, with the relative melanin content amounting to 77.5 ± 1.6% (*p* < 0.001). The relative melanin content of zebrafish embryos treated with YGB at a concentration of 400 μg/mL was 77.3 ± 1.0% (*p* < 0.001), which indicated that ginseng berries could significantly inhibit the melanogenesis of zebrafish. In addition, both RGB and YGB were able to inhibit the tyrosinase activity of zebrafish (*p* < 0.01). The 400 μg/mL YGB tyrosinase activity was determined as 87.370 ± 1.744, which was a highly significant difference (*p* < 0.001) compared with the blank group.

## 3. Materials and Methods

### 3.1. Materials

The red and yellow ginseng berries were collected from the test base of Changchun Agricultural Expo Park in July 2023 and were identified as yellow and red berries of the genus Ginseng in the family of Araliaceae by researcher Zhang Hao from the Institute of Specialties of the Chinese Academy of Agricultural Sciences.

Deoxycholic acid (batch no.: S30218), Arbutin (batch no.: S30884), and levodopa (batch no.: S20191) were purchased from Shanghai Yuanye Biotechnology Co., Ltd. (Shanghai, China). 2,2′-azino-bis (3-ethylbenzothiazoline-6-sulfonic acid) ABTS; 2,2-diphenyl-1-picrylhydrazyl (DPPH); and Potassium persulfate (K_2_O_8_S_2_) were purchased from Shanghai Aladdin Biochemical Technology Co., Ltd. (Shanghai, China). The B16-F10 cell and HaCaT cell were purchased from Procell life Science and Technology Co., Ltd. (Wuhan, China). High-glucose Dulbecco’s Modified Eagle Medium (DMEM); Roswell Park Memorial Institute (RPMI1640) and Fetal Bovine Serum (FBS) were purchased from Gibco BRL (New York, NY, USA).

### 3.2. Extraction and Preparation of Ginseng Berry

The ginseng berries of two berry colors were vacuum dried at 60 °C and then crushed through an 80-mesh sieve. In total, 50 g of the ginseng berry was taken, placed in a round-bottomed flask, added with double-distilled water (material–liquid ratio 1:40 g/mL), continuously heated and refluxed for 120 min, filtered while hot, and the filtrate was concentrated under reduced pressure at 45 °C to 200 mL, vacuum freeze-dried to obtain the RGB and YGB, and placed in a refrigerator at 4 °C for spare.

### 3.3. Chemical Analysis and Compositional Characterization of Ginseng Berry Based on UHPLC-Q-Exactive-MS

#### 3.3.1. Sample Composition Analysis Processing

The sample preparation procedure of Ginseng berry is the same as mentioned in the Section 3.2, which were redissolved with chromatographic methanol–water (8:2) and filtered by the 0.22 μm Teflon microporous filter membrane. Then the filtrate was transferred to a 10 mL volumetric flask and kept in a refrigerator at 4 °C until used.

#### 3.3.2. UHPLC-Q-Exactive-MS Conditions

Chromatographic conditions: Ascentis^®^ Express C_18_ column (3.0 mm × 500 mm, 2.7 μm); mobile phases 0.1% formic-acid–water solution (A), acetonitrile (B), gradient elution (0–5 min, 19%B; 5–12 min, 19–28%B; 12–22 min, 28–40%B; 22–24 min, 40–85%B; 24–25 min, 85–19%B; 25–30 min, 19%B) at a flow rate of 0.2 mL/min; the column temperature was 35 °C; the injection volume was 5 μL.

Mass spectrometry conditions: The ion source was an electrospray ionization (ESI) ion source. The ion source parameters were set as follows: sheath gas flow rate of 40 Arb, auxiliary gas flow rate of 12 Arb, scanning-gas flow rate of 1 Arb. The capillary voltage was set to −3.5 Kv, the capillary temperature was 333 °C, and the ion detection mode was negative ion mode. The scanning modes were Full MS/dd-MS^2^, Full MS with a resolution of 70,000, and dd-MS^2^ with a resolution of 35,000; the scanning range was *m*/*z* 100–1500, and the collision energy was 30 eV.

### 3.4. Antioxidant Capacity Assay

#### 3.4.1. DPPH-Scavenging Activity Assay

In total, 1 mg of DPPH powder was weighed precisely, and methanol was added and fixed to 25 mL to obtain the DPPH-working solution. RGBs and YGBs were diluted to 0.025, 0.5, 0.1, 0.2, 0.4, 0.8, 1, and 2 mg/mL by gradient. In total, 100 μL of the extracts and 100 μL of the DPPH-working solution were allowed to stand in the dark at room temperature for 30 min in three parallel groups, and the absorbance was measured at 517 nm [28,29]. The clearance was calculated according to the following formula:

$$I(\%)=\frac{A0-A2+A1}{A0}\times 100$$

A0: distilled water + DPPH-working solution

A1: ginseng berry sample + methanol

A2: ginseng berry sample + DPPH-working solution

#### 3.4.2. ABTS-Scavenging Activity Assay

RGBs and YGBs were diluted in a gradient to 0.025, 0.5, 0.1, 0.2, 0.4, 0.8, 1, and 2 mg/mL. In total, 100 μL of the extracts were allowed to stand in the dark for 6 min with 100 μL of the ABTS-working solution at room temperature [30,31,32]. The scavenging rate was calculated according to the following formula:

$$I(\%)=\frac{A1}{A0}\times 100$$

A0: PBS + ABTS-working solution

A1: ginseng berry sample + ABTS-working solution

#### 3.4.3. Hydroxyl Radical Assay

The hydroxyl radical assay was performed according to a previous study, with minor optimization [33]. A solution of 5.0 mM FeSO_4_, 5.0 mM ethanolic salicylic acid, and 3 mM H_2_O_2_ was mixed and was reacted for 30 min at 37 °C [34]. The absorbance of the sample was recorded at 510 nm.

#### 3.4.4. FRAP-Scavenging Activity Assay

The FRAP-scavenging activity assay was performed according to a previous study, with minor optimization [35]. Briefly, 200 µL of diluted FRAP solution and 50 µL of sample solutions were mixed in a microplate and incubated for 10 min at 37 °C. The absorbance of the sample was recorded at 592 nm. The antioxidant capacity is expressed as the mass concentration of ferrous sulfate (mg/mL), using ferrous sulfate solution as the standard curve.

### 3.5. Quantification of Chemical Composition of Ginseng Berry

With reference to the quantitative method of total flavonoids, the standard curve was plotted using rutin as the standard [36]. Take 1 mL of ginseng berry dilution, add 1 mL of 5% NaNO_2_ and let it stand for 6 min, then add 1 mL of 10% Al (NO_3_)_3_ and let it stand for 6 min, then add 10 mL of 4% NaOH, and finally, fix the volume with deionized water to 25 mL. The absorbance of the sample was recorded at 510 nm.

With reference to the determination of total saponin content, ginsenoside Re was used as the standard to draw the standard curve [35]. In total, 1 mL of ginseng berry extract was added with 0.2 mL of vanillin and 0.8 mL of perchloric acid, evaporated to dryness at 60 °C, and then added with 5 mL of glacial acetic acid for volume setting. Substitute the absorbance value into the standard curve to calculate the ginsenoside content of the sample.

Referring to the method for the determination of total polysaccharide content, a standard curve was drawn with glucose as the standard [35]. In total, 1 mL of berry extract solution was added with 1 mL of 5% phenol and then 5 mL of sulfuric acid. The absorbance was measured at 490 nm.

Referring to the method for determination of total polyphenol content, the berry extract solution was oxidized with Folin–Ciocalteu reagent, and then the reaction was neutralized with sodium carbonate solution [37]. Absorbance was measured at 765 nm.

### 3.6. Inhibition of Tyrosinase Activity by Ginseng Berry Color Samples

In total, 50 μL L-DOPA (1 mg/mL) and 80 μL PBS (pH = 6.5) were shaken and mixed well, and the reaction was carried out at a constant temperature of 37 °C for 10 min. in total, 10 μL of the sample solutions (RGB and YGB 2.5, 5, 10, 20, 30, 40, and 50 mg/mL), and 10 μL of tyrosinase solution (800 U/mL) were added, and the reaction was shaken and mixed well. Incubate at 37 °C for 5 min. Absorbance was measured at 475 nm, and three sets of parallel tests were performed [34].

### 3.7. HaCaT and B16-F10 Cell Assays with Ginseng Berry

#### 3.7.1. RGB and YGB Concentration Screening and UVB Radiation Dose Screening

HaCaT cells and B16-F10 cells were spread in 96-well plates at 1 × 10^4^. RGBs and YGBs were diluted to 0, 50, 100, 200, and 400 μg/mL with cell culture medium when the cells grew to 80% fusion state after 24 h. Cell viability was detected after 24 h using CCK8 reagent.

HaCaT cells were spread in 96-well plates at 1 × 10^4^. UVB irradiation was performed when the cells grew to 80% fusion state after 24 h. The cells were treated with UVB irradiation. UVB UV radiation screening was performed at 100, 200, 300, 400, 500, 600, and 700 mJ, and cell survival was detected after 24 h using CCK8 reagent.

#### 3.7.2. Detection of Intracellular Indicators of Antioxidant Damage

HaCaT cells were spread in 6-well plates at 1 × 10^6^. When the cells grew to 80% fusion state after 24 h, they were incubated with RGB and YGB (50, 200, and 400 μg/mL) for 4 h and washed twice with PBS. After covering PBS for UVB irradiation of 400 mJ, the cells were collected by continuing incubation with drugs for 24 h. The supernatant was collected by centrifugation at 5000 r/min for 10 min. BCA protein assay kit was used to determine the protein concentration. The levels of SOD activity and MDA content in the cells were determined according to the kit instructions.

#### 3.7.3. Evaluation of Whitening Efficacy of RGB and YGB on B16-F10 Cells

The relative content of melanin was determined by NaOH lysis. B16-F10 cells were spread in 6-well plates at 1 × 106 and divided into blank group, model group, positive control group [38] (1.5 µM arbutin), and drug administration group (50, 200, 400 μg/mL). Cell lysis was performed with PMSF, cells were scraped off with a cell scraper and collected for centrifugation at 12,000 r/min, 4 °C for 10 min. The supernatant was transferred to a new centrifuge tube, and NaOH solution containing 10% DMSO was added to the precipitate, which was lysed for 1 h at 80 °C in a water bath. After the melanin was dissolved, it was vortexed thoroughly, and the absorbance was measured at 475 nm in three parallel groups. The supernatant was taken, 0.1% L-DOPA was added, and the tyrosinase assay was carried out using the levodopa oxidation method (L-DOPA). The sample was incubated at 37 °C for 1 h, and the absorbance was measured at 405 nm in three parallel groups [39].

### 3.8. Evaluation of RGBs and YGBs for Anti-UVB Radiation and Whitening Efficacy in Zebrafish

#### 3.8.1. Determination of UVB Radiation Dose to Zebrafish Embryos

Wild-type zebrafish were used, and the water temperature was maintained at (28.5 ± 0.5) °C. Sixty healthy zebrafish embryos at 8 hpf after fertilization were taken, 10 in each group, and irradiated with UVB lamps at 100, 200, 300, 400, 500, and 600 mJ to observe the growth status of the zebrafish as well as the survival rate.

#### 3.8.2. UVB-Induced Oxidative Damage Staining of ROS in Zebrafish In Vivo

AB embryonic zebrafish with healthy development at 9 hpf after fertilization were taken for incubation and divided into blank group, model group, and administration group (RGBs and YGBs were diluted with zebrafish embryo culture water at 50, 200, and 400 μg /mL) in each concentration of solution for 4 h. The zebrafish embryos were incubated for 72 h in an incubator at 28 °C, with the UVB-induced ROS oxidative damage staining in vivo. Except for the blank group, the zebrafish embryos in each group were irradiated with 400 mJ UVB and incubated at 28 °C incubator for 72 h. Each group consisted of 10 embryos, and three parallel groups were set up. The development of zebrafish embryos in each group was observed, and the drug solution was changed once a day. After 72 h of drug administration, the zebrafish larvae in each group were randomly selected and incubated with DCFH-DA fluorescent probe at 28 °C for 60 min, protected from light, and the stained area of zebrafish was observed under a fluorescence microscope; the results were analyzed statistically by Image-J.

#### 3.8.3. Evaluation of Zebrafish-Whitening Efficacy by RGB and YGB

Eight hpf healthy zebrafish embryos were taken for incubation, grouped as in 4.6.2. The zebrafish were collected after 72 h. Sodium deoxycholate and abrasive beads were added, and the tissues were broken and centrifuged. The supernatant was added with L-DOPA and incubated at 37 °C for 1 h. The absorbance was determined at 405 nm. The precipitate after tissue fragmentation and centrifugation was lysed by adding NaOH containing 10% DMSO and incubated at 80 °C for 1 h. The absorbance was measured at 475 nm [40].

### 3.9. Data Processing and Multivariate Analysis

The LC-MS spectra were viewed using Xcalibur, and the LC-MS data were subjected to peak extraction, peak optimization, and retention time alignment by XCMS I. The heat map analysis of the data was performed in R language (R-4.2.3). The resulting mass spectrometry data were subjected to principal component analysis (PCA) and orthogonal partial least squares discriminant analysis (OPLS-DA) using SIMCA 17.0 to obtain information about the compounds with variable importance projection VIP values >1 and to comprehensively screen for the two compounds with berry color differences. Data were processed using the statistical software SPSS 22.0, and data were expressed as x ± s. Histograms were produced using Graphpad prism 9.3.0 software. Fluorescence intensity analysis was performed using Image J (1.54 j) software.

## 4. Conclusions

In this study, UHPLC-Q-Exactive MS combined with multivariate statistical analysis was used to identify and analyze the constituents of the two fruit colors of ginseng berries and identify the corresponding 31 differential compounds. The content of total ginsenosides in YGB was higher than that in RGB. Feruloyltartaric acid, Ovalitenone, and Garcimangosone D compounds were higher in RGB than in YGB. The in vitro antioxidant capacity assay revealed that RGB had more free-radical-scavenging capacity and better antioxidant activity. On the contrary, YGB had stronger in vitro tyrosinase inhibition than RGB. Total saponins were found to be higher in YGB than RGB by quantitative determination of constituents, and total flavonoids, total polysaccharides, and total polyphenols were higher in RGB than in YGB. Both ginseng berries were able to reduce the accumulation of lipid peroxides MDA; increase SOD content, CAT activity, and GSH-P_X_ activity; inhibit ROS levels; and effectively alleviate UVB-induced oxidative stress. Both ginseng fruits can reduce melanin content and inhibit tyrosinase production. In addition, zebrafish biology verified that RGB was superior to YGB in anti-aging skincare and YGB was superior to RGB in whitening skincare. This not only elucidates the chemical composition and skincare efficacy of the two berry colors of ginseng but also provides a basis for the cultivation and application of ginseng in later stages.

## Figures and Tables

**Table 1:** Annotation of differential compounds UHPLC-Q-Exactive-MS/MS analysis from two-color ginseng berries samples.

NO.	t/min	Extract *m*/*z*	Metabolites	Formula	Monoisotopic Mass	Adducts	Mass Error (mDa)	FC	Log_2_(FC)
1	1.03	371.1203	Feruloyltartaric acid	C_14_H_14_O_9_	326.0632	M + FA-H	12	0.74	−0.43
2	1.1	337.0898	3-O-p-Coumaroylquinic acid	C_16_H_18_O_8_	338.0996	M-H	6	2.11	1.08
3	1.2	551.0994	Quercetin 3-O-(6″acetyl-glucoside)	C_23_H_22_O_13_	506.1054	M + FA-H	3	3.32	1.73
4	1.36	337.0707	Ovalitenone	C_19_H_14_O_6_	338.0784	M-H	0	0.39	−1.37
5	1.39	385.0787	Cichoriin	C_15_H_16_O_9_	340.0788	M + FA-H	7	2.05	1.04
6	1.62	391.1012	Garcimangosone D	C_19_H_20_O_9_	392.1101	M-H	3	0.21	−2.22
7	10.96	845.4908	Ginsenoside Rg1	C_42_H_72_O_14_	800.4916	M + FA-H	5	1.78	0.83
8	11.23	991.5483	Ginsenoside Re	C_48_H_82_O_18_	946.5495	M + FA-H	2	2.40	1.26
9	11.38	969.5128	2′-O-Acetyl-akebia saponin D	C_49_H_78_O_19_	970.5131	M-H	8	6.17	2.63
10	15.94	1031.543	Ginsenoside mRe	C_51_H_84_O_21_	1032.5499	M-H	1	2.50	1.32
11	16	987.5531	Ac-ginsenoside Re	C_50_H_84_O_19_	988.5601	M-H	1	2.43	1.28
12	16.02	1055.539	Pubescenoside D	C_53_H_84_O_21_	1056.5499	M-H	3	7.48	2.90
13	19.58	845.4908	Ginsenoside Rf	C_42_H_72_O_14_	800.4916	M + FA-H	8	2.79	1.48
14	20.54	815.4803	Ginsenoside F3	C_41_H_70_O_13_	770.4810	M + FA-H	6	2.05	1.04
15	21.56	829.4957	Ginsenoside Rg2	C_42_H_72_O_13_	784.4967	M + FA-H	5	3.05	1.61
16	21.85	815.4798	Ginsenoside F5	C_41_H_70_O_13_	770.4810	M + FA-H	6	1.75	0.81
17	21.92	1107.593	Ginsenoside Rb1	C_54_H_92_O_23_	1108.6023	M-H	1	2.49	1.32
18	21.94	1131.595	Quinquenoside R1	C_56_H_94_O_24_	1150.6129	M-H20-H	0	4.00	2.00
19	22.32	1193.596	Ginsenoside mRb1	C_57_H_94_O_26_	1194.6027	M-H	1	2.31	1.21
20	22.56	1123.59	Ginsenoside Rc	C_53_H_90_O_22_	1078.5918	M + FA-H	4	2.37	1.24
21	22.59	1077.583	Ginsenoside Rb2	C_53_H_90_O_22_	1078.5918	M-H	1	2.59	1.37
22	22.62	1191.578	Diosgenin 3- [glucosyl-(1->6)-glucosyl-(1->4)-rhamnosyl-(1->4) [rhamnosyl-(1->2)]-glucoside]	C_57_H_92_O_26_	1192.5871	M-H	3	2.58	1.37
23	23	1163.585	Ginsenoside mRc	C_56_H_92_O_25_	1164.5922	M-H	1	2.34	1.23
24	23.06	1119.593	Ginsenoside Rs1	C_55_H_92_O_23_	1120.6023	M-H	1	2.32	1.22
25	24.64	1163.585	Ginsenoside mRb2	C_56_H_92_O_25_	1164.5922	M-H	0	2.29	1.19
26	24.66	1119.595	Ginsenoside Rs2	C_55_H_92_O_23_	1120.6023	M-H	0	2.29	1.19
27	24.7	991.5481	Ginsenoside Rd	C_48_H_82_O_18_	946.5495	M + FA-H	5	1.74	0.80
28	24.72	969.5121	3′-O-Acetyl-akebia saponin D	C_49_H_78_O_19_	970.5131	M-H	7	2.55	1.35
29	25.12	1031.543	Ginsenoside mRd	C_51_H_84_O_21_	1032.5499	M-H	1	1.54	0.62
30	25.18	987.5521	AcO-Ginsenoside Rd	C_50_H_84_O_19_	988.5601	M-H	0	1.53	0.61
31	27.78	829.4958	Ginsenoside Rg3	C_42_H_72_O_13_	784.4967	M + FA-H	5	1.35	0.44

**Table 2:** Determination of antioxidant capacity and content of red and yellow berries

		RGB	YGB
Antioxidant capacity (IC50)	DPPH	0.576 ± 1.052	0.657 ± 0.263
	ABTS	1.044 ± 0.548	1.050 ± 0.846
	OH	0.023 ± 1.320	0.049 ± 0.985
	FRAP	1.481 ± 0.895	1.646 ± 1.223
Ingredient content (%)	Total flavonoids	3.772 ± 0.078	1.879 ± 0.050
	Total saponins	17.199 ± 0.901	26.921 ± 1.352
	Total polysaccharide	22.675 ± 0.632	19.021 ± 2.044
	Total polyphenols	1.925 ± 0.079	0.999 ± 0.044

**Table 3:** Determination of oxidative damage indexes of ginseng red and yellow berries.

	SOD (U/L)	MDA (nmol/mL)	CAT (U/mg Prot)	GSH-Px (U/mg Prot)
Blank group	26.254 ± 1.691 ***	23.691 ± 1.194 ***	43.214 ± 3.056 ***	62.403 ± 4.465 ***
Model Groups	11.131 ± 3.382	42.116 ± 0.269	9.542 ± 4.258	37.380 ± 2.940
RGB 50 μg/mL	17.400 ± 1.888 *	37.272 ± 0.955 ***	12.806 ± 0.876	35.139 ± 3.738
RGB 200 μg/mL	21.129 ± 2.693 ***	32.836 ± 1.166 ***	17.242 ± 1.575 ***	44.366 ± 0.345
RGB 400 μg/mL	27.593 ± 1.651 ***	29.430 ± 0885 ***	28.914 ± 3.045 ***	58.141 ± 2.596 ***
YGB 50 μg/mL	15.535 ± 1.052	38.180 ± 0.205 **	10.598 ± 2.763	33.805 ± 1.148
YGB 200 μg/mL	18.468 ± 0.986 **	35.743 ± 0.425 ***^,#^	15.623 ± 0.944 ***	42.582 ± 2.770 ***
YGB 400 μg/mL	22.112 ± 1.808 ***^,#^	33.239 ± 0.990 ***^,###^	22.815 ± 1.042 ***^,##^	49.341 ± 0.743 ***^,#^

Note: Compared with the model group * *p* < 0.05; ** *p* < 0.01; *** *p* < 0.001. Compared with RGB ^#^ *p* < 0.05; ^##^ *p* < 0.01; ^###^ *p* < 0.001. Superoxide dismutase (SOD); Malondialdehyde (MDA); Catalase From Micrococcus Lysodeikticus (CAT); Glutathione peroxidase (GSH-Px).

## Data Availability

The datasets used and/or analyzed during the present study are available from the corresponding author upon reasonable request.
